# Machine learning-based nomogram for mortality risk stratification in cirrhotic patients with sepsis: a single-center predictive model

**DOI:** 10.3389/fmed.2025.1684527

**Published:** 2025-10-21

**Authors:** Xing-Cheng Zhang, Bo-Wen Li, Xi-Qun Lei, Nan-Bing Shan, Jun-Ping Wei, Zhong-Hua Lu, Yun Sun

**Affiliations:** ^1^The First Department of Critical Care Medicine, The Second Affiliated Hospital of Anhui Medical University, Hefei, Anhui, China; ^2^Department of Critical Care Medicine, Fuyang Infectious Disease Clinical College of Anhui Medical University, Fuyang, Anhui, China; ^3^Department of Critical Care Medicine, Bozhou People's Hospital, Bozhou1, Anhui, China; ^4^Department of Respiratory Medicine, Linquan County People's Hospital, Fuyang, Anhui, China

**Keywords:** liver cirrhosis, sepsis, LASSO regression, mortality, nomogram, prediction

## Abstract

**Objective:**

To develop and validate a nomogram-based predictive model for in-hospital mortality among patients with liver cirrhosis complicated by sepsis, and to evaluate its predictive accuracy.

**Methods:**

Clinical data were retrospectively collected from patients diagnosed with liver cirrhosis and sepsis who were admitted to the Fuyang Infectious Disease Clinical College of Anhui Medical University between January 2018 and July 2025. Patients were classified into the Survivor group or the Non-survivor group. The dataset was randomly divided into a training set (70%) and a validation set (30%). Potential predictors were identified through univariate and multivariate logistic regression analyses, and a predictive model was subsequently developed using Lasso regression. The model was visualized as a nomogram, and its performance was rigorously evaluated using receiver operating characteristic (ROC) curves, calibration plots, and decision curve analysis (DCA) to assess its clinical utility.

**Results:**

A total of 264 patients were enrolled in this study. Among the 188 patients in the training set, 54 (28.7%) died during hospitalization, while 21 out of 76 patients (27.6%) in the validation set experienced in-hospital mortality. Multivariate logistic regression analysis identified alcoholic cirrhosis, Child-Pugh score, mechanical ventilation, TBiL and HR as independent predictors of in-hospital mortality (all *P* < 0.05). The nomogram model demonstrated robust predictive performance, with ROC analysis showing an area under the curve (AUC) of 0.81 (95% CI: 0.75–0.81) in the training set and 0.83 (95% CI: 0.73–0.92) in the validation set. Calibration plots revealed that the model's predictions closely aligned with the ideal reference line. DCA showed that the model provided significant clinical net benefit across a wide range of threshold probabilities.

**Conclusion:**

The nomogram model developed using Lasso regression appears to demonstrate promising predictive potential for in-hospital mortality in patients with liver cirrhosis complicated by sepsis. This tool may offer valuable support for clinical decision-making and could potentially aid in guiding early interventions for patients identified as higher risk.

## 1 Introduction

Sepsis is defined as a life-threatening organ dysfunction caused by a dysregulated host response to infection. Epidemiological evidence indicates that it accounts for approximately 250,000 annual deaths in the United States, with associated healthcare expenditures reaching nearly 62 billion US dollars. In China, the reported 30-day all-cause mortality rate among septic patients is 29.5%, compared to 24.4% in Europe and North America (1–3). Approximately one-quarter of sepsis cases progress to septic shock, a severe clinical syndrome associated with multiple organ dysfunction, particularly involving the cardiovascular system, kidneys, and liver. When sepsis affects three or more organ systems, the mortality rate may rise to approximately 60% (4–6). According to clinical definitions, septic shock is characterized by persistent circulatory and metabolic abnormalities accompanied by organ dysfunction. Given its high incidence and associated mortality, septic shock remains a significant challenge in clinical medicine (7, 8).

Liver cirrhosis is a chronic liver disease caused by various etiological factors, characterized pathologically by diffuse hepatic fibrosis and pseudolobular formation (9). Compared with individuals without liver cirrhosis, patients with liver cirrhosis demonstrate a 4- to 5-fold increased risk of infection due to acquired immunodeficiency resulting from hypersplenism, which leads to reduced white blood cell counts and impaired production of immune proteins (10, 11). Upon the onset of infection, cirrhotic patients face an elevated risk of developing sepsis, accompanied by significantly higher mortality. Evidence indicates that the mortality rate among patients with liver dysfunction complicated by sepsis is approximately four times greater than that observed in patients with sepsis alone, with an estimated 30% of cirrhotic patients succumbing within 1 month following infection (12). Therefore, early prognostic evaluation is essential for guiding clinical decision-making and optimizing therapeutic strategies in patients with liver cirrhosis complicated by sepsis. Although several studies have identified risk factors associated with in-hospital mortality following septic shock, the distinct epidemiological features, clinical manifestations, and mechanisms of immune dysregulation in cirrhotic patients with sepsis remain poorly understood. Moreover, due to the complexity and heterogeneity of their clinical conditions, patients with liver cirrhosis are frequently excluded from randomized controlled trials, leading to a relative lack of research focused on this high-risk population. To address this knowledge gap, the present study specifically targeted patients with liver cirrhosis complicated by sepsis, thereby minimizing confounding effects arising from multiple etiologies. By analyzing baseline clinical data, key predictors of in-hospital mortality were identified, and a nomogram-based predictive model was developed to facilitate rapid and accurate prognostic assessment for this vulnerable patient cohort.

## 2 Materials and methods

### 2.1 Research subjects

A retrospective analysis was conducted on the clinical data of patients diagnosed with liver cirrhosis complicated by sepsis who were admitted to the Fuyang Infectious Disease Clinical College of Anhui Medical University between January 2018 and July 2025. These patients were randomly divided into either the training set or the validation set in a 7:3 ratio.

#### 2.1.1 Inclusion criteria

(1) Diagnosis fulfilled the criteria for liver cirrhosis as specified in the “Evidence-Based Clinical Practice Guidelines for Liver Cirrhosis 2020” (13); (2) Fulfillment of the diagnostic criteria for sepsis or septic shock as defined in the “Surviving Sepsis Campaign: International Guidelines for Management of Sepsis and Septic Shock 2021” (14); (3) Age ≥ 18 years.

#### 2.1.2 Exclusion criteria

(1) Hospitalization duration less than 24 h; (2) Presence of severe hematological disorders, immunodeficiency, or malignant tumors; (3) Premature withdrawal of treatment by family members; (4) Incomplete or inadequate medical documentation.

### 2.2 Ethics

This study was conducted in accordance with the principles of medical ethics and was approved by the Institutional Ethics Committee (Approval No. 20241012075). Written informed consent was obtained from the legal guardians or family members of all participants prior to the initiation of any treatment or clinical procedures.

### 2.3 Treatment

Upon admission, all patients were managed in accordance with the Surviving Sepsis Campaign guidelines and the Evidence-Based Clinical Practice Guidelines for Liver Cirrhosis 2020 (14, 15). The treatment protocol included anti-inflammatory therapy, respiratory support, fluid resuscitation, antimicrobial therapy, administration of vasoactive agents to maintain circulatory stability, and other supportive interventions as clinically indicated.

### 2.4 Data collection

Baseline clinical data were collected, including demographic characteristics (gender, age), Admitted to the ICU, infection site, progression of liver cirrhosis, and associated complications. Additionally, the Acute Physiology and Chronic Health Evaluation II (APACHE II) score, Quick Sequential Organ Failure Assessment (qSOFA) score, and Child-Pugh score were recorded on the first day of hospital admission. Routine physiological and biochemical parameters were also obtained, including respiratory rate (RR), heart rate (HR), mean arterial pressure (MAP), procalcitonin (PCT) level, C-reactive protein (CRP), white blood cell (WBC) count, platelet (PL) count, alanine aminotransferase (ALT), aspartate aminotransferase (AST), blood urea nitrogen (BUN), serum creatinine (Cr), fibrinogen (FIB) level, and serum albumin. Information regarding the administration of vasoactive drugs, mechanical ventilation, hormone therapy, and continuous renal replacement therapy (CRRT) was also documented.

### 2.5 Short-term prognosis

The in-hospital mortality rate among patients with liver cirrhosis complicated by sepsis was analyzed as the primary outcome. Patients who died during hospitalization were assigned to the Non-survivor group (*n* = 75), including those who chose to withdraw life-sustaining treatment before death, while those who survived were categorized into the Survivor group (*n* = 189).

### 2.6 Statistical analysis

Statistical analyses were conducted using SPSS version 26.0 and R version 4.4.1. Quantitative data are expressed as mean ± standard deviation; for non-normally distributed data, the median and interquartile range (IQR) was reported. Categorical variables are presented as frequencies and percentages. Inter-group comparisons of continuous variables were performed using the independent samples t-test or the Mann-Whitney U-test, depending on the data distribution. Categorical variables were analyzed using the chi-square test or Fisher's exact test. The dataset was randomly divided into a training set and a validation set at a ratio of 7:3. Potential risk factors associated with in-hospital mortality were identified through Lasso regression combined with logistic multivariate analysis, and a predictive model was subsequently developed. Once the regression equation was established, the model was visualized using a nomogram, and its performance was evaluated by means of the receiver operating characteristic (ROC) curve. The area under the ROC curve (AUC) was calculated to quantify the model's discriminatory ability. Model calibration was assessed graphically via a calibration curve. Additionally, decision curve analysis (DCA) was employed to evaluate the clinical utility of the model. A *p-value* < 0.05 was considered statistically significant.

## 3 Results

### 3.1 Comparison of baseline characteristics

No statistically significant differences were observed between the Non-survivor group and the Survivor group with respect to demographic and clinical characteristics, including gender, age, admitted to the ICU, length of hospital stay, presence of bloodstream infection, abdominal cavity infection, pulmonary infection, urinary tract infection, or skin infection. Additionally, no significant differences were found in comorbidities such as hypertension, diabetes, COPD, CHD, duration of liver cirrhosis, portal hypertension, hepatic encephalopathy, hepatocellular carcinoma, acute liver failure, chronic liver failure, multiple organ failure, hepatitis B, hepatitis C, and other etiological factors. Similarly, no significant differences were observed in severity assessment scores, including qSOFA and APACHE II scores, or in the utilization of vasoactive drugs, hormone therapy, CRRT, and albumin administration. Laboratory parameters such as ALT, AST, DBiL, BUN, blood glucose, serum potassium, serum sodium, WBC count, neutrophil percentage, hemoglobin level, PLT count, PT, INR, FIB, PTA, CRP, MAP, and RR did not show statistically significant differences between groups (*p* > 0.05). However, several key variables demonstrated statistically significant differences (*p* < 0.05), including the incidence of spontaneous bacterial peritonitis, occurrence of esophageal and gastric variceal bleeding, Alcoholic, Child-Pugh score, requirement for mechanical ventilation, TBiL, Cr, PCT concentration, HR, and body temperature (see Table 1).

**Table 1:** Comparison of outcomes.

**Variables**	**Survivors (*n* = 189)**	**Non-survivors (*n* = 75)**	**All patients (*n* = 264)**	***P***
Gender				0.501
Female	43 (22.8)	20 (26.7)	63 (23.9)	
Male	146 (77.2)	55 (73.3)	201 (76.1)	
Age [years, mean (SD)]	55.8 (12.7)	57.6 (12.5)	56.3 (12.6)	0.315
Admitted to the ICU				1.000
No	63 (33.3)	25 (33.3)	88 (33.3)	
Yes	126 (66.7)	50 (66.7)	176 (66.7)	
Length of hospital stay [days, median (IQR)]	9 (4.15)	8 (4.5, 17.5)	9 (4.16)	0.407
Bloodstream infection				0.976
No	149 (78.8)	59 (78.7)	208 (78.8)	
Yes	40 (21.2)	16 (21.3)	56 (21.2)	
Abdominal cavity infection				0.216
No	68 (36)	21 (28)	89 (33.7)	
Yes	121 (64)	54 (72)	175 (66.3)	
Pulmonary infection				0.419
No	137 (72.5)	58 (77.3)	195 (73.9)	
Yes	52 (27.5)	17 (22.7)	69 (26.1)	
Urinary tract infection				0.717
No	183 (96.8)	72 (96)	255 (96.6)	
Yes	6 (3.2)	3 (4)	9 (3.4)	
Skin infection				1.000
No	186 (98.4)	74 (98.7)	260 (98.5)	
Yes	3 (1.6)	1 (1.3)	4 (1.5)	
Hypertension				0.382
No	164 (86.8)	68 (90.7)	232 (87.9)	
Yes	25 (13.2)	7 (9.3)	32 (12.1)	
Diabetes				0.207
No	163 (86.2)	60 (80)	223 (84.5)	
Yes	26 (13.8)	15 (20)	41 (15.5)	
COPD				0.625
No	186 (98.4)	73 (97.3)	259 (98.1)	
Yes	3 (1.6)	2 (2.7)	5 (1.9)	
CHD				0.278
No	184 (97.4)	71 (94.7)	255 (96.6)	
Yes	5 (2.6)	4 (5.3)	9 (3.4)	
Course of liver cirrhosis [years, median(IQR)]	2 (0.9, 5)	3 (1, 5.5)	2 (1.5)	0.520
Portal hypertension				0.894
No	100 (52.9)	39 (52)	139 (52.7)	
Yes	89 (47.1)	36 (48)	125 (47.3)	
Spontaneous bacterial peritonitis				0.013
No	110 (58.2)	31 (41.3)	141 (53.4)	
Yes	79 (41.8)	44 (58.7)	123 (46.6)	
Hepatic encephalopathy				0.083
No	150 (79.4)	52 (69.3)	202 (76.5)	
Yes	39 (20.6)	23 (30.7)	62 (23.5)	
Esophageal and gastric variceal bleeding				0.030
No	150 (79.4)	50 (66.7)	200 (75.8)	
Yes	39 (20.6)	25 (33.3)	64 (24.2)	
Hepatocellular carcinoma				0.677
No	126 (66.7)	52 (69.3)	178 (67.4)	
Yes	63 (33.3)	23 (30.7)	86 (32.6)	
Acute liver failure				0.788
No	164 (86.8)	66 (88)	230 (87.1)	
Yes	25 (13.2)	9 (12)	34 (12.9)	
Chronic liver failure				0.304
No	138 (73)	50 (66.7)	188 (71.2)	
Yes	51 (27)	25 (33.3)	76 (28.8)	
Multiple organ failure				1.000
No	185 (97.9)	73 (97.3)	258 (97.7)	
Yes	4 (2.1)	2 (2.7)	6 (2.3)	
Alcoholic				< 0.001
No	159 (84.1)	37 (49.3)	196 (74.2)	
Yes	30 (15.9)	38 (50.7)	68 (25.8)	
Hepatitis B				0.448
No	81 (42.9)	36 (48)	117 (44.3)	
Yes	108 (57.1)	39 (52)	147 (55.7)	
Hepatitis C				0.460
No	174 (92.1)	71 (94.7)	245 (92.8)	
Yes	15 (7.9)	4 (5.3)	19 (7.2)	
Others				0.931
No	172 (91)	68 (90.7)	240 (90.9)	
Yes	17 (9)	7 (9.3)	24 (9.1)	
qSOFA score [score, median (IQR)]	1 (0.2)	1 (0.2)	1 (0.2)	0.206
APACHE-II score	16 (12, 21)	18 (13, 26)	17 (12, 23)	0.083
Childpugh score [score, median(IQR)]				< 0.001
Grade A	48 (25.4)	8 (10.7)	56 (21.2)	
Grade B	76 (40.2)	15 (20)	91 (34.5)	
Grade C	65 (34.4)	52 (69.3)	117 (44.3)	
Vasoactive drugs				0.710
No	93 (49.2)	35 (46.7)	128 (48.5)	
Yes	96 (50.8)	40 (53.3)	136 (51.5)	
Hormone				0.305
No	176 (93.1)	67 (89.3)	243 (92)	
Yes	13 (6.9)	8 (10.7)	21 (8)	
Mechanical ventilation				< 0.001
No	146 (77.2)	39 (52)	185 (70.1)	
Yes	43 (22.8)	36 (48)	79 (29.9)	
CRRT				0.170
No	166 (87.8)	61 (81.3)	227 (86)	
Yes	23 (12.2)	14 (18.7)	37 (14)	
Albumin [g/L, mean (SD)]	29 (6.2)	27.6 (5)	28.6 (5.9)	0.079
ALT [U/L, median (IQR)]	38 (24, 64)	34 (20, 52.5)	36 (22.8, 62.2)	0.198
AST [U/L, median(IQR)]	62(38,120)	62(38.8,131)	62(38.4,121.2)	0.825
TBiL [μmol/L, median(IQR)]	39.1 (21.2, 110.4)	150.2 (85.1, 213.4)	63.9 (24.8, 165.7)	< 0.001
DBiL [μmol/L, median(IQR)]	41 (13.7, 121.9)	64.1 (25.3, 146.8)	46.2 (15.4, 132.7)	0.068
BUN [mmol/L, median (IQR)]	11.4 (6.6, 17.6)	12 (8.2, 21.7)	11.4 (7.2, 19)	0.077
Cr [μmol/L, median(IQR)]	106 (64, 192)	135 (91.5, 218)	110 (67, 201.2)	0.049
Blood sugar [μmol/L, median (IQR)]	5.6 (4.4, 7.5)	5.2(4.2, 7.1)	5.5 (4.2, 7.3)	0.189
Blood potassium [mmol/L, median (IQR)]	3.9 (3.5, 4.5)	4.1 (3.6, 4.8)	4 (3.5, 4.6)	0.212
Blood sodium [mmol/L, median (IQR)]	134.3 (128.5, 137.6)	133.5 (127.6, 138.1)	134.1 (128.3, 137.6)	0.516
WBC [×10^9^/L, median (IQR)]	9 (5.6, 14.6)	9.2 (4.8, 14.8)	9 (5.5, 14.7)	0.646
neutrophil percentage [%, median (IQR)]	83.5 (73, 89.3)	82.1 (73.5, 90.1)	82.8 (73.2, 89.5)	0.802
Hemoglobin [g/L, mean (SD)]	102.5 (26.9)	104.8 (23.8)	103.1 (26)	0.518
PLT [×10^9^/Lmedian (IQR)]	77 (44, 131)	79 (44, 132.5)	78 (44, 132.2)	0.732
PT [seconds, median (IQR)]	16.8 (14, 21)	17.8 (15.2, 24)	17.1 (14.3, 21.6)	0.144
INR [median (IQR)]	1.5 (1.2, 1.9)	1.5 (1.3, 2.1)	1.5 (1.2, 1.9)	0.212
FIB [g/L, median (IQR)]	2.2 (1.5, 3.5)	2.1 (1.5, 2.9)	2.2 (1.5, 3.3)	0.587
PTA [%, median (IQR)]	51.8 (37.8, 70)	48.3 (33.1, 60)	50.2 (36.6, 68.5)	0.215
CRP [mg/L, median (IQR)]	70.9 (22.6, 116.1)	49.6 (27.1, 115.5)	63.7 (25.8, 116)	0.853
PCT [ng/mL, median (IQR)]	3.7 (0.7, 18)	15.7 (3.1, 32.9)	4.9 (0.8, 23.2)	0.002
MAP [mmHg, median (IQR)]	76 (66, 88)	79 (68, 95)	77 (67, 90)	0.139
HR [beats per minute, median (IQR)]	90 (78, 102)	82 (75, 97.5)	89 (78, 102)	0.031
RR [breaths per minute, median (IQR)]	20 (18, 21)	19 (18, 22)	20 (18, 21.2)	0.646
Body temperature [°C, median(IQR)]	36.6 (36.5, 36.9)	36.5 (36.3, 36.8)	36.6 (36.4, 36.8)	0.027

COPD, Chronic obstructive pulmonary disease; CHD, Coronary heart disease; APACHE-II, Acute Physiology and Chronic Health Evaluation II; CRRT, Continuous renal replacement therapy; RR, respiratory rate; HR, heart rate; MAP, mean arterial pressure; PCT, procalcitonin; CRP, C-reactive protein; WBC, white blood cell; PLT, platelet; ALT, alanine aminotransferase; AST, aspartate aminotransferase; TBiL, Total bilirubin; DBiL, Direct bilirubin; BUN, blood urea nitrogen; Cr, creatinine; PT, Prothrombin time; INR, International standard ratio; FIB, ibrinogen; PTA, Prothrombin activity.

### 3.2 Baseline comparison between the training set and validation set

The baseline characteristics, including demographic data (gender, age), clinical parameters (Admitted to the ICU, length of hospital stay), types of infection (bloodstream infection, abdominal cavity infection, pulmonary infection, urinary tract infection, skin infection), and comorbidities (hypertension, diabetes, COPD, CHD) were compared between the Non-survivor group and the Survivor group. Additionally, liver-related clinical features—including duration of liver cirrhosis (years), presence of portal hypertension, spontaneous bacterial peritonitis, hepatic encephalopathy, esophageal and gastric variceal bleeding, hepatocellular carcinoma, acute liver failure, chronic liver failure, and multiple organ failure—as well as etiological factors such as Alcoholic, hepatitis B, hepatitis C, and other causes—were analyzed. Severity assessment scores, including qSOFA, APACHE II, and Child-Pugh scores, along with treatment interventions such as administration of vasoactive drugs, hormone therapy, mechanical ventilation, CRRT, and albumin infusion, were also evaluated. Laboratory parameters encompassing ALT, AST, TBiL, DBi, BUN, Cr, blood glucose, serum potassium, WBC count, neutrophil percentage, hemoglobin level, albumin, PLT count, PT, INR, FIB, PTA, CRP, PCT, MAP, RR, and body temperature were assessed. No statistically significant differences were observed in these variables between the training set and validation set (*p* > 0.05), except for serum sodium levels, which demonstrated a statistically significant difference between the two groups (*p* < 0.05) (see Table 2).

**Table 2:** Baseline comparison of the training set and validation set.

**Variables**	**Validation set (*n* = 76)**	**Training set (*n* = 188)**	**All patients (*n* = 264)**	***P***
Gender				0.965
Female	18 (23.7)	45 (23.9)	63 (23.9)	
Male	58 (76.3)	143 (76.1)	201 (76.1)	
Age [years, mean (SD)]	58.2 (14.2)	55.6 (11.9)	56.3 (12.6)	0.124
Admitted to the ICU				0.442
No	28 (36.8)	60 (31.9)	88 (33.3)	
Yes	48 (63.2)	128 (68.1)	176 (66.7)	
Length of hospital stay [days, median (IQR)]	9.5 (4, 16.2)	8 (4,16)	9 (4, 16)	0.915
Bloodstream infection				0.481
No	62 (81.6)	146 (77.7)	208 (78.8)	
Yes	14 (18.4)	42 (22.3)	56 (21.2)	
Abdominal cavity infection				0.298
No	22 (28.9)	67 (35.6)	89 (33.7)	
Yes	54 (71.1)	121 (64.4)	175 (66.3)	
Pulmonary infection				0.376
No	59 (77.6)	136 (72.3)	195 (73.9)	
Yes	17 (22.4)	52 (27.7)	69 (26.1)	
Urinary tract infection				1.000
No	74 (97.4)	181 (96.3)	255 (96.6)	
Yes	2 (2.6)	7 (3.7)	9 (3.4)	
Skin infection				0.581
No	76 (100)	184 (97.9)	260 (98.5)	
Yes	0 (0)	4 (2.1)	4 (1.5)	
Hypertension				0.456
No	65 (85.5)	167 (88.8)	232 (87.9)	
Yes	11 (14.5)	21 (11.2)	32 (12.1)	
Diabetes				0.499
No	66 (86.8)	157 (83.5)	223 (84.5)	
Yes	10 (13.2)	31 (16.5)	41 (15.5)	
COPD				0.628
No	74 (97.4)	185 (98.4)	259 (98.1)	
Yes	2 (2.6)	3 (1.6)	5 (1.9)	
CHD				0.720
No	73 (96.1)	182 (96.8)	255 (96.6)	
Yes	3 (3.9)	6 (3.2)	9 (3.4)	
Course of liver cirrhosis [years, median (IQR)]	3 (0.7, 6)	2 (1, 5)	2 (1,5)	0.684
Portal hypertension				0.589
No	42 (55.3)	97 (51.6)	139 (52.7)	
Yes	34 (44.7)	91 (48.4)	125 (47.3)	
Spontaneous bacterial peritonitis				0.072
No	34 (44.7)	107 (56.9)	141 (53.4)	
Yes	42 (55.3)	81 (43.1)	123 (46.6)	
Hepatic encephalopathy				0.061
No	64 (84.2)	138 (73.4)	202 (76.5)	
Yes	12 (15.8)	50 (26.6)	62 (23.5)	
Esophageal and gastric variceal bleeding				0.442
No	60 (78.9)	140 (74.5)	200 (75.8)	
Yes	16 (21.1)	48 (25.5)	64 (24.2)	
Hepatocellular carcinoma				0.424
No	54 (71.1)	124 (66)	178 (67.4)	
Yes	22 (28.9)	64 (34)	86 (32.6)	
Acute liver failure				0.369
No	64 (84.2)	166 (88.3)	230 (87.1)	
Yes	12 (15.8)	22 (11.7)	34 (12.9)	
Chronic liver failure				0.792
No	55 (72.4)	133 (70.7)	188 (71.2)	
Yes	21 (27.6)	55 (29.3)	76 (28.8)	
Multiple organ failure				0.187
No	76 (100)	182 (96.8)	258 (97.7)	
Yes	0 (0)	6 (3.2)	6 (2.3)	
Alcoholic				0.658
No	55 (72.4)	141 (75)	196 (74.2)	
Yes	21 (27.6)	47 (25)	68 (25.8)	
Hepatitis B				0.237
No	38 (50)	79 (42)	117 (44.3)	
Yes	38 (50)	109 (58)	147 (55.7)	
Hepatitis C				0.421
No	69 (90.8)	176 (93.6)	245 (92.8)	
Yes	7 (9.2)	12 (6.4)	19 (7.2)	
Others				0.144
No	66 (86.8)	174 (92.6)	240 (90.9)	
Yes	10 (13.2)	14 (7.4)	24 (9.1)	
qSOFA score [score, median (IQR)]	1 (0, 2)	1 (0, 2)	1 (0, 2)	0.347
APACHE-II score	17 (11.8, 22.2)	17 (12, 24)	17 (12, 23)	0.332
Childpugh score [score, median (IQR)]				0.896
Grade A	17 (22.4)	39 (20.7)	56 (21.2)	
Grade B	27 (35.5)	64 (34)	91 (34.5)	
Grade C	32 (42.1)	85 (45.2)	117 (44.3)	
Vasoactive drugs				0.817
No	36 (47.4)	92 (48.9)	128 (48.5)	
Yes	40 (52.6)	96 (51.1)	136 (51.5)	
Hormone				0.304
No	72 (94.7)	171 (91)	243 (92)	
Yes	4 (5.3)	17 (9)	21 (8)	
Mechanical ventilation				0.826
No	54 (71.1)	131 (69.7)	185 (70.1)	
Yes	22 (28.9)	57 (30.3)	79 (29.9)	
CRRT				0.299
No	68 (89.5)	159 (84.6)	227 (86)	
Yes	8 (10.5)	29 (15.4)	37 (14)	
Albumin [g/L, mean (SD)]	28 (25.5, 30.8)	28.9 (24,33)	28.4 (24.3, 32.2)	0.621
ALT [U/L, median (IQR)]	34.5 (21.8, 53)	37.5 (23, 64.2)	36 (22.8, 62.2)	0.392
AST [U/L, median (IQR)]	61.5 (37.2, 80)	62 (38.9, 137.8)	62 (38.4, 121.2)	0.243
TBiL [μmol/L, median (IQR)]	41.8 (22.7, 195.4)	66.6 (26.6, 161.3)	63.9 (24.8, 165.7)	0.676
DBiL [μmol/L, median (IQR)]	45.8 (17.9, 166.7)	46.2 (15.2, 112.2)	46.2 (15.4, 132.7)	0.494
BUN [mmol/L, median (IQR)]	10.8 (6.4, 18.3)	11.6 (7.9, 19)	11.4 (7.2, 19)	0.500
Cr [μmol/L, median(IQR)]	105.5 (61, 188.2)	115.5 (69.5, 207)	110 (67, 201.2)	0.249
Blood sugar [μmol/L, median(IQR)]	5.3 (4.2, 6.8)	5.6(4.4,7.6)	5.5 (4.2, 7.3)	0.272
Blood potassium [mmol/L, median (IQR)]	4 (3.5, 4.5)	4 (3.5, 4.7)	4 (3.5, 4.6)	0.705
Blood sodium [mmol/L, median (IQR)]	132.5 (126.9, 136.9)	134.3 (129.9, 138)	134.1 (128.3, 137.6)	0.046
WBC [×10?/L, median(IQR)]	8 (5, 15.1)	9.3 (5.6, 14.6)	9 (5.5, 14.7)	0.398
neutrophil percentage [%, median (IQR)]	83.4 (74, 90.2)	82.7 (72.7, 88.9)	82.8 (73.2, 89.5)	0.367
Hemoglobin [g/L, mean (SD)]	103.7 (24.8)	102.9 (26.6)	103.1 (26)	0.822
PLT [×10?/Lmedian (IQR)]	76 (47.2, 129.2)	79.5 (43, 133)	78 (44, 132.2)	0.952
PT [seconds, median (IQR)]	16.9 (14.4, 21.8)	17.2 (14.3, 21.6)	17.1 (14.3, 21.6)	0.990
INR [median (IQR)]	1.4 (1.3, 1.9)	1.5 (1.2, 1.9)	1.5 (1.2, 1.9)	0.826
FIB [g/L, median (IQR)]	2.2 (1.5, 3.4)	2.2 (1.5, 3.2)	2.2 (1.5, 3.3)	0.608
PTA [%, median (IQR)]	51.4 (36.5, 64.1)	50 (36.6, 70)	50.2 (36.6, 68.5)	0.674
CRP [mg/L, median (IQR)]	49.5 (20.3, 105.6)	71.4 (27.8, 118.1)	63.7 (25.8, 116)	0.253
PCT [ng/mL, median (IQR)]	5.5 (1.4, 19)	4.4 (0.7, 23.4)	4.9 (0.8, 23.2)	0.930
MAP [mmHg, median (IQR)]	76.5 (66.8, 89.5)	77 (67, 90)	77 (67, 90)	0.979
HR [beats per minute, median (IQR)]	88 (77.5, 100)	89 (78, 103.2)	89 (78, 102)	0.251
RR [breaths per minute, median (IQR)]	20 (18, 22)	19 (18, 21)	20 (18, 21.2)	0.206
Body temperature [°C, median (IQR)]	36.6 (36.5, 36.8)	36.6 (36.3, 36.8)	36.6 (36.4, 36.8)	0.222
Outcome				0.859
Survivors	55 (72.4)	134 (71.3)	189 (71.6)	
Nonsurvivors	21 (27.6)	54 (28.7)	75 (28.4)	

### 3.3 Data screening

**Figure 1:**
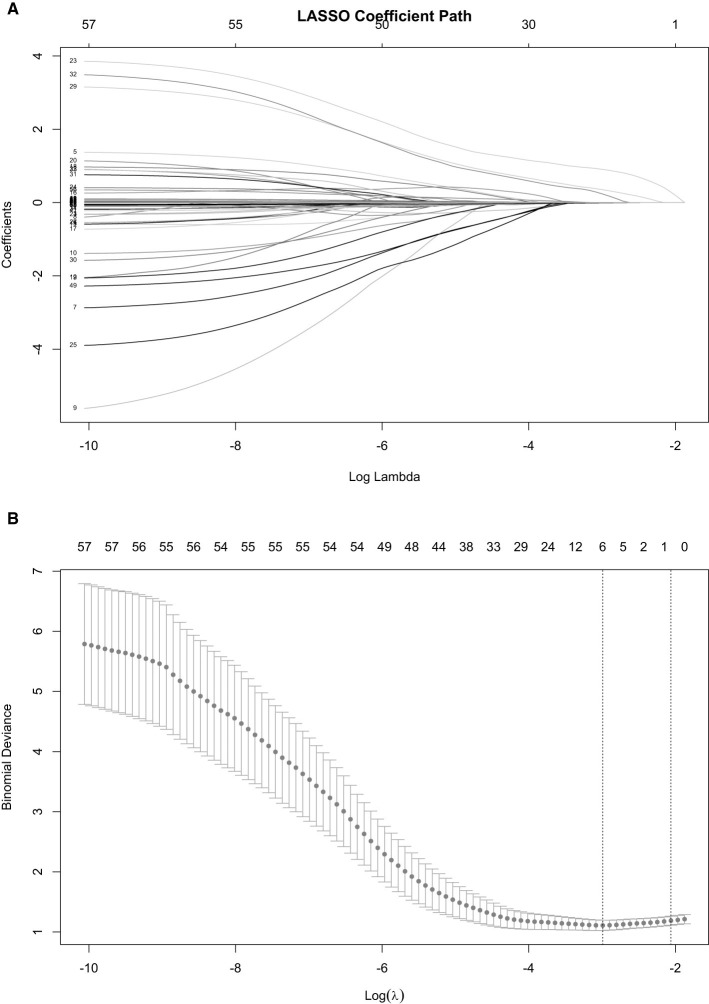
Schematic diagram of Lasso regression. **(A)** illustrates the relationship between the logarithm of the regularization parameter (LogLambda) and the corresponding coefficient values (Coefficients). Each colored curve represents the variation trend of a specific variable's coefficient as the Lambda value changes. **(B)** displays the relationship between the logarithm of u [Log(u)] and the binomial deviance. The red line indicates the overall trend of this relationship, while the 95% confidence interval is represented by the gray shaded area. Vertical dashed lines correspond to two key u-values that are of particular interest in model selection.

LASSO regression analysis was performed on potential predictive factors, and the significantly associated variables identified included Alcoholic cirrhosis, Child-Pugh score, requirement for mechanical ventilation, TBiL level, PCT concentration, and HR (Figure 1).

The factors screened out by Lasso regression were included in the multi-factor stage and analyzed through logistic multi-factor regression. Alcoholic (OR = 3.703, 95% CI: 1.68–8.315, *P* = 0.001), Childpugh score (OR = 2.155, 95% CI: 1.283–3.786, *P* = 0.005), mechanical ventilation (OR = 2.658, 95% CI: 1.212–5.928, *P* = 0.015), TBiL (OR = 1.004, 95% CI: 1–1.007, *P* = 0.028), PCT (OR = 1.012, 95% CI: 0.998–1.027, *P* = 0.084), HR (OR = 0.969, 95% CI: 0.947–0.989, *P* = 0.005) is a significant risk factor (see Table 3).

**Table 3:** Multivariate logistic results.

**Variable**	**B**	**S.E**	**Wald**	***P***	**OR**	**95% CI**	
						**Lower**	**Upper**
Alcoholic	1.309	0.406	3.224	0.001	3.703	1.68	8.315
Childpugh score	0.768	0.274	2.799	0.005	2.155	1.283	3.786
Mechanical ventilation	0.978	0.403	2.428	0.015	2.658	1.212	5.928
TBiL	0.004	0.002	2.193	0.028	1.004	1	1.007
PCT	0.012	0.007	1.727	0.084	1.012	0.998	1.027
HR	−0.031	0.011	−2.828	0.005	0.969	0.947	0.989

### 3.4 Construction of nomogram prediction model

**Figure 2:**
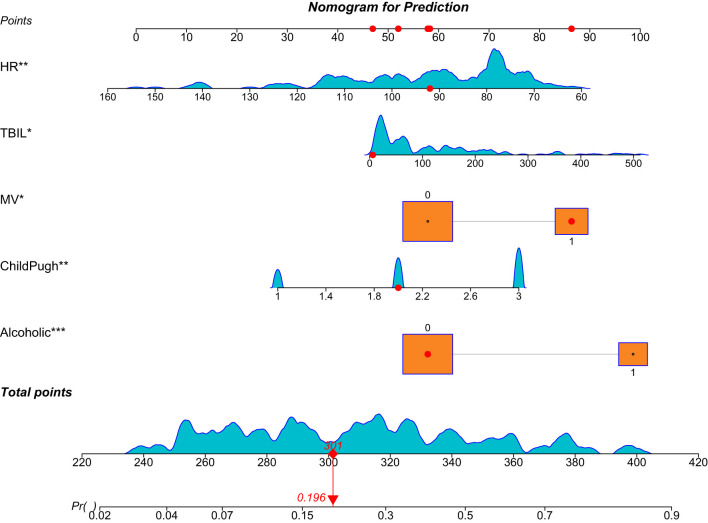
This figure shows the nomogram of the prediction model. Each predictor in the nomogram (such as Alcoholic cirrhosis, Child-Pugh score, mechanical ventilation, TBiL, and HR) is assigned a specific score based on its value range. These individual scores are summed to yield a “total score,” which is then mapped to a corresponding risk probability on the “Diagnostic Prediction Probability” scale. This allows for the estimation of the patient's in-hospital mortality risk at the given threshold. *, **, *** represent three different levels of significance: less than 0.05, less than 0.01, and less than 0.001.

Based on the results of Lasso regression, models were established using Alcoholic cirrhosis, Child-Pugh score, mechanical ventilation, TBiL, and HR as predictors. The equations were visualized through a nomogram, as shown in Figure 2.

### 3.5 Model evaluation

The ROC curves of the model in the training set and the validation set demonstrate its discriminatory ability. In the training set, the AUC of the model was 0.81 (95% CI: 0.75–0.81), and in the validation set, it was 0.83 (95% CI: 0.73–0.92). These results indicate that the diagnostic performance of the model is stable across different datasets, with strong generalization ability.

**Figure 3:**
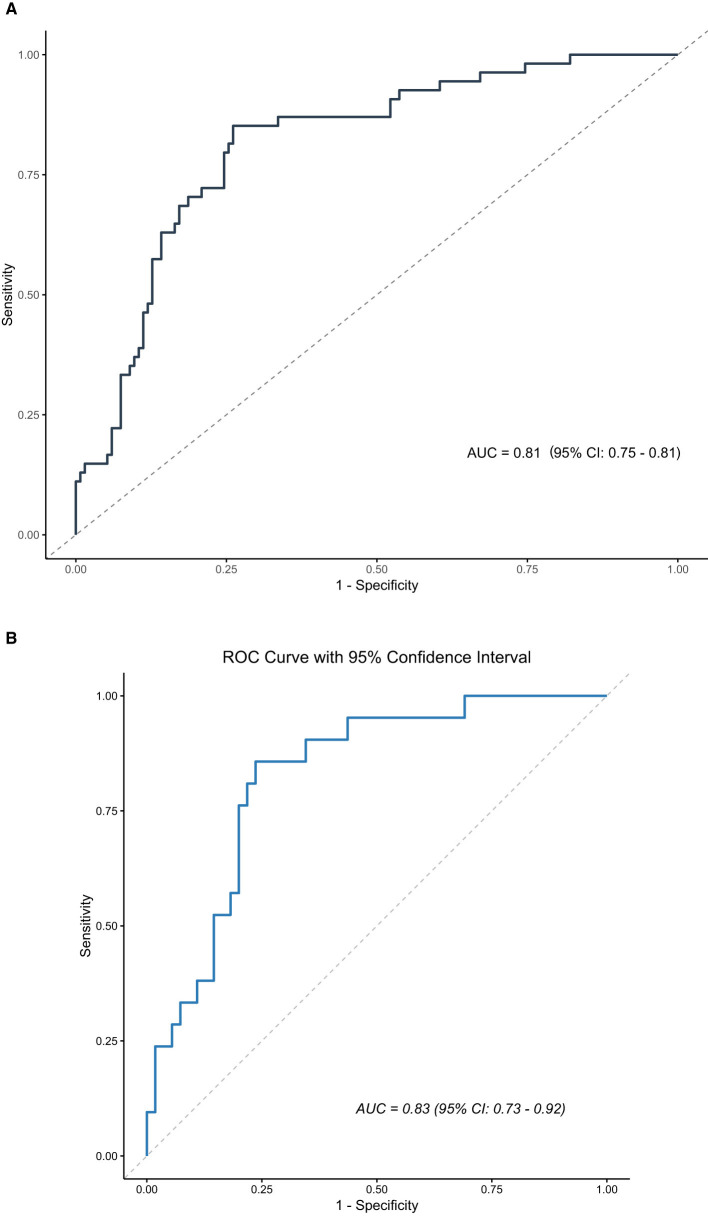
Receiver operating characteristic (ROC) curves for the model in **(A)** the training set and **(B)** the validation set. The x-axis shows 1-specificity (false-positive rate) and the y-axis shows sensitivity (true-positive rate). The area under the curve (AUC) summarizes discrimination; values closer to 1 indicate better performance.

The calibration curves of the predictive model for both the training and validation sets are presented in Figure 3. In both sets, the horizontal axis represents the predicted risk probability, and the vertical axis shows the observed proportion of outcomes. The ideal calibration curve, which aligns with the diagonal reference line, reflects perfect prediction accuracy.

**Figure 4:**
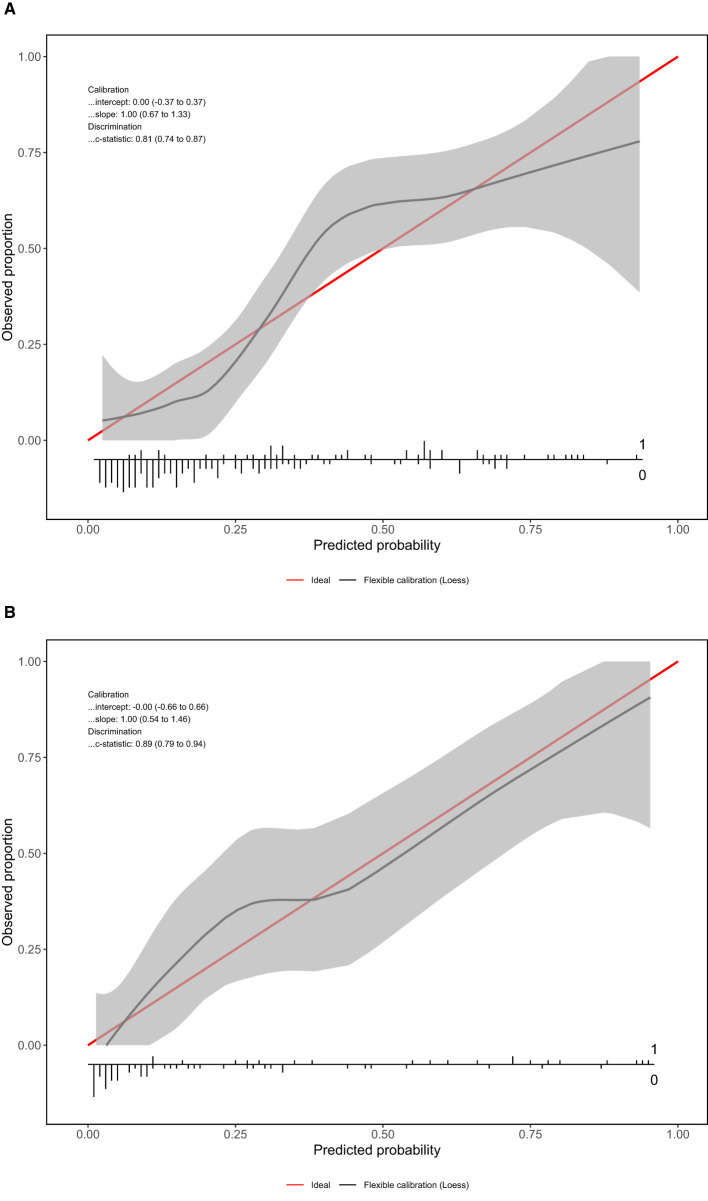
Calibration curves for the model in **(A)** the training set and **(B)** the validation set. The x-axis shows predicted probability and the y-axis shows observed outcome frequency.

In the training set, the calibration curve exhibited a slope of 1.00 (95% CI: 0.67–1.33) and an intercept of 0.00 (95% CI: −0.37 to 0.37), indicating excellent agreement between predicted and observed probabilities. The c-statistic for the training set was 0.81 (95% CI: 0.74–0.87), further confirming the model's strong discriminatory performance. Similarly (Figure 4A), in the validation set, the calibration curve demonstrated a slope of 1.00 (95% CI: 0.54–1.46) and an intercept of −0.00 (95% CI: −0.66 to 0.66), showing a near-perfect alignment with the ideal line. The c-statistic for the validation set was 0.89 (95% CI: 0.79–0.94), indicating robust performance and high reliability across both datasets (Figure 4B).

**Figure 5:**
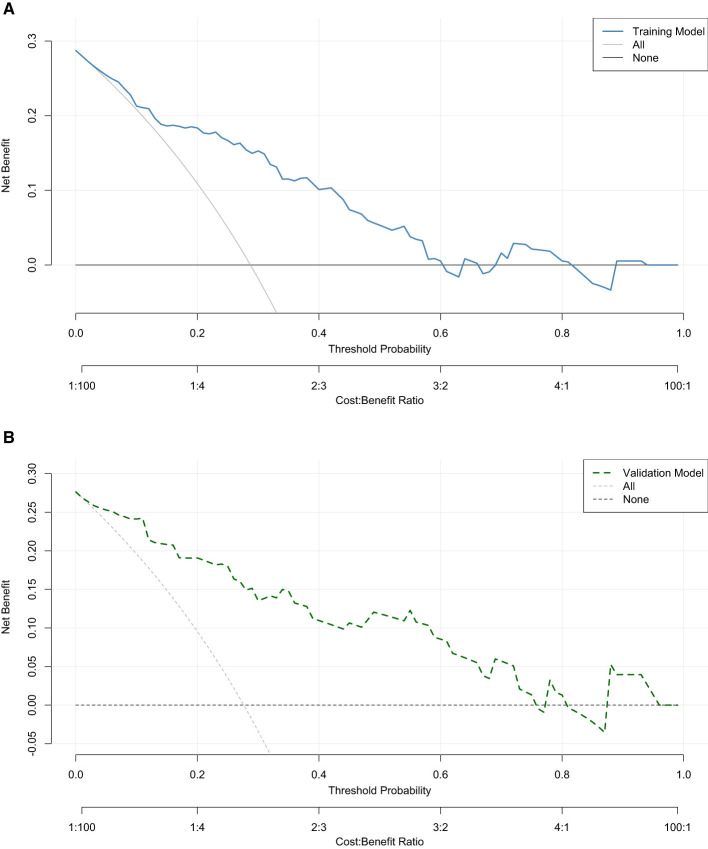
Decision curve analysis (DCA) for the model in **(A)** the training set and **(B)** the validation set. The y-axis shows net benefit across threshold probabilities on the x-axis; higher curves indicate greater clinical utility compared with treat-all and treat-none strategies.

The decision curve analysis (DCA) for both the training and validation sets is shown in Figure 5. In both sets, the horizontal axis represents the threshold probability, and the vertical axis shows the net benefit of using the predictive model to guide clinical decisions. In the training set (Figure 5A), the decision curve demonstrates that the model provides significant clinical net benefit across a wide range of threshold probabilities, including low to moderate risk scenarios, compared to the “All” and “None” strategies. The net benefit of the model improves as the threshold probability increases, with the curve indicating that the model is clinically useful for guiding decisions regarding treatment allocation. Similarly, in the validation set (Figure 5B), the decision curve shows that the model continues to provide substantial net benefit across various threshold probabilities, reinforcing its clinical utility. The model outperforms the “All” and “None” strategies, suggesting its potential for improving patient outcomes through more precise risk stratification.

## 4 Discussions

Studies indicate that infection is a critical contributor to adverse outcomes in patients with liver cirrhosis. Research data suggest that approximately 15% to 35% of hospitalized patients with liver cirrhosis develop infections during their inpatient stay (16, 17). Despite early initiation of anti-infective therapy, infection recurrence remains common, primarily due to immune dysfunction and the presence of multi-drug resistant pathogens (18). When infection progresses to sepsis, the mortality risk for patients with liver cirrhosis increases significantly (19). According to existing literature, the mortality rate among patients with liver cirrhosis complicated by sepsis is approximately four times higher than that of patients with uncomplicated liver cirrhosis, and their hospital stays are substantially prolonged (20). Furthermore, a bidirectional relationship exists between liver cirrhosis and sepsis: liver cirrhosis predisposes patients to sepsis, while sepsis-induced organ dysfunction and elevated mortality further worsen the underlying liver disease (10). Therefore, early prognostic assessment and timely clinical interventions are essential for managing patients with liver cirrhosis and concurrent sepsis. In this study, an in-hospital mortality rate of 28.41% was observed among patients with liver cirrhosis complicated by sepsis in the training cohort, highlighting the substantial mortality risk following disease onset. Multivariate logistic regression analysis identified several independent predictors of in-hospital mortality, including a history of alcoholic cirrhosis, Child-Pugh score, requirement for mechanical ventilation, TBiL levels, and HR. These factors collectively influence clinical outcomes, underscoring the importance of close monitoring of these parameters by clinicians.

Historically, chronic liver disease associated with hepatitis B virus infection was more prevalent. However, with advancements in the management of viral hepatitis and changes in lifestyle patterns, the incidence of alcoholic liver disease has been rising rapidly. Data from the United States indicate that alcohol has become the second most common cause of liver cirrhosis, accounting for approximately 20% to 25% of all cases (21). The findings of this study are consistent with these trends: among 264 patients diagnosed with cirrhosis and concurrent sepsis, hepatitis B-related cirrhosis was the most common type (55.7%), followed by alcoholic cirrhosis (25.8%). Notably, a large hospital-based cohort study conducted in India reported that the proportion of alcoholic liver disease reached as high as 39.4% (22). Regional data from Asia also indicate a continuous increase in the mortality rate among patients with alcoholic liver cirrhosis in recent years (23–25). In this study, the in-hospital mortality rate among patients with alcoholic cirrhosis complicated by sepsis was as high as 50.7%, which may be partially attributed to concomitant hepatitis B infection. PCT has been widely recognized as a valuable biomarker for assessing the severity of infection in patients with liver cirrhosis and sepsis. During the progression or systemic spread of infection, the immune system releases a significant amount of inflammatory mediators, such as cytokines and inflammatory proteins, which stimulate the production and release of PCT. Lazzarotto et al. (26) demonstrated that both CRP and PCT are reliable indicators of bacterial infection among hospitalized cirrhotic patients, with elevated levels showing a strong correlation with short-term mortality. However, the results of the multivariate analysis in this study did not reveal a statistically significant association between PCT levels and in-hospital mortality.

Studies have demonstrated that during sepsis, the liver's capacity to clear endotoxins is compromised, leading to impaired bile secretion and consequently intrahepatic cholestasis. Concurrently, inflammatory mediators can disrupt the bile acid transport function of basolateral membrane proteins in hepatocytes and bile ducts, thereby contributing to elevated serum TBil levels (27, 28). Accumulating evidence indicates a strong association between hyperbilirubinemia and adverse clinical outcomes in patients with cirrhosis or critical illness (29), with increased conjugated bilirubin serving as a significant prognostic marker for mortality in both acute and chronic liver failure (30). Our findings support this evidence. In the context of liver function assessment, the Child-Pugh scoring system is widely recognized for its comprehensive evaluation, incorporating key parameters such as portal pressure, hepatic synthetic function, and the presence of complications. Higher scores are indicative of more severe portosystemic shunting, greater gastrointestinal congestion, and poorer prognosis. A systematic review and meta-analysis comparing the prognostic accuracy of the Child-Pugh and Model for End-Stage Liver Disease (MELD) scores in patients with cirrhosis revealed comparable predictive performance in most clinical settings (31). Furthermore, the need for mechanical ventilation represents a crucial prognostic factor influencing outcomes in patients with cirrhosis complicated by sepsis. Patients in the non-survivor group are more likely to develop respiratory failure due to pulmonary infection or acute respiratory distress syndrome (ARDS), reflecting a more severe disease state and a significantly higher mortality risk (32).

Indicators such as PT and D-dimer exhibit potential predictive value in evaluating the severity and prognosis of patients with sepsis. Research suggests that the “inflammation-coagulation interaction” plays a pivotal role in coagulation dysfunction associated with sepsis. The excessive release of inflammatory mediators can rapidly activate the procoagulant system, thereby triggering both endogenous and exogenous coagulation pathways. This activation leads to substantial consumption of coagulation factors, often reflected by prolonged PT (33). However, univariate analysis in this study did not detect statistically significant differences in coagulation parameters such as PT, which may be attributed to the limited sample size.

Nevertheless, it remains crucial to monitor for possible coagulation disorders and secondary hyperfibrinolysis in critically ill patients. Common complications of liver cirrhosis include ascites, sepsis, hepatic encephalopathy (HE), hepatocellular carcinoma (HCC), spontaneous bacterial peritonitis (SBP), hepatorenal syndrome (HRS), and esophageal or gastric variceal bleeding. These complications frequently require hospitalization and are associated with increased morbidity, mortality, and healthcare expenditures. Among these, HE is a prevalent and potentially reversible neuropsychiatric disorder in patients with liver cirrhosis, with an incidence rate reaching up to 50% (34). Moreover, studies (35) indicate that cirrhotic patients complicated by HRS have higher short-term mortality rates. Sepsis-induced organ dysfunction is partially attributed to inadequate tissue perfusion and abnormal cellular metabolism (36). Persistent inflammatory responses and impaired resolution of sepsis may further exacerbate organ damage and potentially lead to HRS (37). In this study, multivariate analysis did not reveal statistically significant associations between complications such as SBP, HE, and esophageal or gastric variceal bleeding and clinical outcomes. The authors hypothesize that this may be related to the clinical characteristics of the enrolled population: not all patients were critically ill, and most were newly diagnosed during hospitalization, relatively young, and had fewer comorbidities. Consequently, their stronger immune response and compensatory capacity might have influenced the distribution and severity of complications.

Albumin, as a key indicator of hepatic synthetic function, has been widely acknowledged for its clinical relevance in evaluating the prognosis of patients with liver cirrhosis, as fluctuations in its levels are closely associated with patient outcomes (38). However, relying solely on a single biomarker for prognostic assessment presents notable limitations. Recent studies have demonstrated that the lactate/albumin ratio (LAR), as a composite biomarker, offers distinct advantages in predicting disease progression and adverse outcomes. Accumulating evidence indicates that LAR not only serves as an effective predictor of multiple organ failure in patients with severe sepsis but is also significantly correlated with overall mortality (39). A recent large-scale study based on the MIMIC-IV database further confirmed the prognostic value of LAR specifically in patients with liver cirrhosis complicated by sepsis (12). Regarding renal function evaluation, serum creatinine levels upon admission have emerged as a critical prognostic factor. Renal insufficiency is commonly observed in patients with liver cirrhosis, and this comorbidity significantly increases the risk of mortality (40). The underlying pathophysiological mechanisms primarily involve hemodynamic alterations induced by portal hypertension and reduced renal perfusion due to splanchnic vasodilation. When combined with sepsis, systemic inflammatory responses and hemodynamic instability may precipitate acute kidney injury, thereby worsening clinical outcomes (41, 42). Clinical observations have further shown that even minor increases in serum creatinine levels (≥0.3 mg/dL) may indicate impending adverse events (43). Notably, given the altered metabolism of creatinine in patients with liver cirrhosis, it is essential to incorporate additional clinical parameters—such as urine output—into the assessment of renal function to overcome the limitations of relying solely on serum creatinine measurements (44).

Currently, the prognostic value of individual hemodynamic parameters—such as HR and MAP—in predicting mortality among patients with cirrhosis complicated by sepsis remains controversial. A more comprehensive assessment that integrates these hemodynamic indicators with other essential clinical variables is required for accurate risk stratification and outcome prediction. From a pathophysiological standpoint, changes in HR have dual implications: early compensatory tachycardia (>100 beats per minute) is a characteristic feature of the initial septic response, whereas persistent tachycardia indicates an uncontrolled systemic inflammatory reaction and is significantly associated with increased mortality. Late-stage bradycardia may reflect sepsis-induced myocardial depression or circulatory collapse, both of which are typically linked to a poor prognosis. Moreover, autonomic dysfunction, frequently observed in patients with cirrhosis, further reduces HR variability and contributes to elevated mortality risk. With regard to MAP, a decrease below 65 mmHg not only serves as a key diagnostic criterion for septic shock but also represents a common clinical challenge in cirrhotic patients due to pre-existing vasodilation and reduced effective circulating blood volume, often resulting in refractory hypotension. Additionally, the limited effectiveness of fluid resuscitation in this population makes maintaining an adequate MAP particularly difficult, which is closely associated with higher mortality rates. Notably, a narrowed pulse pressure may indicate hypovolemia or diminished cardiac output, thereby further deteriorating the clinical course. Furthermore, multiple factors—including impaired hepatic functional reserve (e.g., Child-Pugh class C or MELD score >20), complications related to portal hypertension (e.g., gastrointestinal bleeding, hepatorenal syndrome), and immune dysfunction (e.g., susceptibility to fungal or multidrug-resistant bacterial infections)—interact synergistically and collectively influence clinical outcomes. Therefore, a multidimensional and multiparametric approach is essential for the accurate prognostic evaluation of patients with cirrhosis and concurrent sepsis.

In the past, various scoring systems have been developed to predict disease progression in patients with liver cirrhosis or sepsis, such as the SOFA, the MELD-Na, and the age-bilirubin-INR-creatinine (ABIC) score (45, 46). With advancements in clinical standards, recent research has increasingly focused on the prognosis of patients suffering from liver cirrhosis complicated by sepsis. However, due to the limited number of cases involving end-stage liver disease, cirrhosis, or liver failure complicated by sepsis, there remains a lack of large-scale, multicenter clinical data to support accurate prognostic evaluation for this specific patient population. Recently, two predictive models based on the MIMIC database were developed to estimate in-hospital mortality among patients with liver cirrhosis and concomitant sepsis. Although these models included a substantial number of cases—primarily from Western countries—they focused exclusively on critically ill patients admitted to the ICU. In contrast, our study included 88 non-ICU-admitted patients with liver cirrhosis complicated by sepsis, among whom the mortality rate reached as high as 33.3%, highlighting the need for increased attention to this subgroup. Moreover, some existing assessment tools or models are not widely adopted in clinical practice due to factors such as high costs, time-consuming implementation, or challenges in acquiring essential information during the early stages of disease (32, 47–49). In this study, we constructed a nomogram-based prediction model to identify risk factors associated with in-hospital mortality in patients with sepsis complicated by liver cirrhosis. The total score of this model ranges from 220 to 420, corresponding to mortality probabilities between 0.02 and 0.9. The model was validated using both ROC curve analysis and calibration curves. The results demonstrated that the AUC value for predicting in-hospital mortality was 0.81 (95% confidence interval: 0.75–0.81) in the training set and 0.83 (95% confidence interval: 0.73–0.92) in the validation set. Further calibration analysis suggested a close correspondence between predicted mortality rates and the ideal reference curve, indicating reasonably strong predictive performance. Decision curve analysis (DCA) further supported the potential clinical value of the model, showing favorable net benefits across most threshold probabilities. The newly developed model presents a potentially useful approach for assessing in-hospital mortality risk in patients with cirrhotic sepsis. It may assist healthcare professionals in visually interpreting how routine laboratory indicators influence outcomes, thereby contributing to the development of more tailored treatment strategies based on individual patient profiles.

In conclusion, this study developed a predictive model using Lasso regression to assess the risk of in-hospital mortality among patients with liver cirrhosis complicated by sepsis. Several limitations of the study should be acknowledged: First, due to the retrospective nature of the hospital-based database, certain clinically relevant variables—such as lactate (50) and D-dimer—were excluded from the analysis because of missing data exceeding 20%, which may introduce potential bias into the results. Second, the prediction model proposed in this study has not yet been externally validated or compared with established scoring systems such as SOFA, MELD, and ABIC. Nevertheless, we applied rigorous inclusion and exclusion criteria to ensure that the data from both the survival and non-survival groups accurately represented real-world clinical scenarios. Finally, as a retrospective observational study, there may be unmeasured confounding factors that could affect the validity of the conclusions. Therefore, further prospective studies are required to validate the predictive performance of this nomogram before it can be implemented in clinical practice.

## Data Availability

The original contributions presented in the study are included in the article/supplementary material, further inquiries can be directed to the corresponding authors.
